# Syphilis and Sycosis

**Published:** 1898-04

**Authors:** George J. Augur


### SYPHILIS AND SYCOSIS.

GEORGE J. AUGUR, M. D.

[Read before *The Organon* and Materia Medica Club of the Bay Cities of California.]

At the request of our President I have attempted to epitomize Hahnemann's views on "Sycosis and Syphilis." Of these two miasms, which Hahnemann designates as causing by far the smaller part of the chronic diseases, we will review the first. This miasm he tells us has produced the fewest chronic diseases, and has only been dominant from time to time, raging with the greatest activity during the French war from the years 1807 to 1814; that the treatment of this disease by allopathic physicians from its supposed alliance to the venereal chancre affection was by Mercury internally, while the excrescences were treated by cauterizing, burning, cutting, and ligation. These excrescences usually manifest themselves on the genitals, and are attended by a sort of gonorrhœa, though not always, appearing several days, weeks or even months after infection; in character soft and spongy, bleeding easily, more rarely dry like warts. That he considers this disease, sycosis, to differ from the common gonorrhœa in that it is constitutional is manifest, for he says: "The miasms of other common gonorrhœas seems not to penetrate the whole organism, but only locally to stimulate the urinary organs. They yield either to a dose or two of fresh Parsley Juice, Cannabis, Cantharides, or Copaiva Balm, according to the difference in the constitution of the individual affected or other ailments attending the disease."

These remedies should be used in the higher or highest potencies in case a psora slumbering in the body of the patient has been developed by means of a strongly affecting, irritating or weakening treatment by allopathic physicians. In such cases secondary gonorrhœas frequently remain, which are only to be cured by anti-psoric treatment. Hahnemann still further emphasizes the difference in the two diseases, in that the treatment of the fig-wart disease by external destruction of the excrescences in no way diminishes the miasm which rules in the whole organism. Nor does the Mercury taken internally affect the disease favorably, being in no way appropriate to sycosis. Besides undermining the general health by this remedy (which is usually given in large doses), similar excrescences then break out in other parts of the body, either whitish, spongy, sensitive, flat elevations in the cavity of the mouth or on the tongue, the palate and the lips, or as large, raised brown tubercles in the axilla, on the neck, on the scalp, etc., or there arise other ailments of the body. The gonorrhœas dependent upon the fig-wart miasma, as well as the excrescences, are cured most surely, most thoroughly, through the internal use of Thuja potentized to the decillioneth degree, and when these have exhausted their action, after fifteen to forty days, use a small dose of Nitric-acid in the same potency, which must be allowed to act as long a time in order to remove the whole sycosis. It is not necessary to use any external application except in the most inveterate and difficult cases, when the large fig-warts may be moistened every day with the pure juice pressed from the green leaves of Thuja mixed with an equal quantity of alcohol. "If the patient was at the same time affected by another chronic ailment, as is usual after the violent treatment of fig-warts by allopathic physicians, then we often find developed psora complicated with sycosis. When the psora, as is often the case, was latent before in the patient, or as might happen, both these miasma are conjoined with syphilis, then it is necessary to come to the assistance of the most afflicted part, the psora," by the appropriate remedy, and then to make use of the remedy for sycosis, before the proper dose of the best preparation of Mercury is given for the syphilis. The same plan of treatment may be continued until a complete cure is effected.

### SYPHILIS.

We now come to the second chronic miasma, which is far more common and widely spread, and has, according to Hahnemann, been the source of many other chronic ailments. In the cure of this disease, syphilis, three states or conditions are to be considered:

*First.* When the initial lesion, the chancre, still remains, or if this has been removed by local applications, the second manifestation of syphilis, the bubo, is present, and this usually only appears after removal of the chancre by external treatment.

*Second.* When the disease is alone without the complication of a second or third miasma, and also without the vicarious symptoms of either the chancre or bubo.

*Third.* When it is complicated with another chronic disease, *i. e.*, with a psora already developed, while the local symptoms of the malady may still be present or removed by local treatment. The evidences of this disease appear as we know after impure coition, usually from seven to fourteen days, first as a small pustule, then changing into a foul ulcer with raised edges. The ulcer, though increasing in size, will remain until the end of a man's natural life if not cured by the indicated homœopathic remedy or removed by the usual allopathic practice of local applications. Furthermore, the chancre is a vicarious index of the syphilitic miasma, and as long as it remains unhealed no secondary symptoms of this venereal disease will appear. Should, however, this local manifestation of the specific constitutional condition be suppressed, and no secondary symptoms be present to indicate whether the disease is cured or simply slumbering, there can be observed a sign in way of a reddish or blue scar at the point where the ulcer had been, showing that the disease still exists in the system.

If, on the other hand, the disease has been really eradicated from the system, as it can be by one dose of the best mercurial preparation within fourteen days, if the condition be as described in the first simple state, then there will exist no discoloration of the skin at the point where the chancre had been.

Even in the second state, in which the chancre and bubo have been driven away through local applications, but there exists no developed psora as a complication, "all outbreaks of the secondary venereal disease may be avoided, and the man may be freed from every trace of the venereal miasma through the before-mentioned simple internal cure effected by a like dose of the above-mentioned mercurial medicine." All signs even in this case of discoloration at the point of the initial sore will have disappeared, because of the action of the internal homœopathic remedy, and because of the absence of psoric complications.

We come now to the third and most difficult form to cure—when the syphilis is complicated with developed psora. This psoric disease may have been manifest in way of chronic ailments at the time the syphilis was contracted, or slumbering in the system may have become aroused by strong allopathic treatment. In either case when the disease is combined with developed psora it is impossible to cure the venereal disease alone. It can only be done by first giving the anti-psoric remedy which is homœopathically best fitted to the prevailing symptoms, and allowing it to act as long as it will, and then possibly a second most suited to the remaining psoric symptoms, allowing it to act until all has been accomplished against the psoric disease which can be at this time. Then the syphilitic condition can be attacked by the best preparation of Mercury, as has been described.

If there should be a three-fold complication in way of three chronic miasms, as very rarely happens—the fig-wart disease with the venereal chancre miasm, and at the same time a developed psora—these cases are to be cured according to the same method, *i. e.*, the psora to be treated first, then one of the two remaining, the symptoms of which was the most prominent at the time, and then the last one.

In closing, I will state that according to Hahnemann psora can only act as a complication to venereal disease when it has been developed, and when it has manifested itself in way of a chronic disease, but not when it is latent and slumbering.

